# What is the best therapeutic approach if the initial glucocorticoids therapy of active Graves’ orbitopathy fails?

**DOI:** 10.1186/1756-6614-6-S2-A24

**Published:** 2013-04-05

**Authors:** Helena Jastrzębska

**Affiliations:** 1 Endocrinology Department of Medical Centre of Postgraduate Education, Warsaw, Poland

## 

Glucocorticoids are the first-line treatment for active moderate-to-severe and sight-threatening Graves’ orbitopathy. Glucocorticoid therapy, by exerting antiinflammatory and immunomodulatory effects, reduces pain, injection and edema of orbital tissues and provides a substantial relief from optic nerve compression. Although efficiency of both oral and intravenous glucocorticoids is proven, the randomized clinical trials revealed that intravenous therapy is more effective and better tolerated. However, 20-40% of patients respond only partially or do not respond to immunosuppressive treatment. The effectiveness of treatment may be improved by proper selection of patients suspected to have beneficial results, i.e. those with a high degree of disease activity, with orbitopathy of recent onset or recent progression. Corticotherapy may cause major side effects which make treatment continuation impossible. Orbital radiotherapy exerts nonspecific antiinflammatory effect and suppresses radiosensitive lymphocytes infiltrating the orbital space. Orbital radiotherapy remains an effective and safe second-line treatment preferably used in association with glucocorticoids. The combined therapy is more effective than either treatment alone. Sight-threatening Graves’ orbitopathy due to compressive optic neuropathy or corneal ulceration requires immediate intervention. High-dose daily intravenous glucocorticoids are the first-line treatment and if the response is absent or poor within 1-2 weeks the orbital decompression should be performed. Corneal breakdown should be additionally treated with topical lubricants, and sometimes, to provide eyelid closure, with blepharorraphy or tarsorraphy. Unsuccessful oral glucocorticoid treatment of active Graves’ orbitopathy should be an indication for orbital radiotherapy, preferably combined with glucocorticoids, or to a 3 month intravenous methylprednisolone course. If intravenous methylprednisolone was the first treatment and the response is not satisfactory, the intravenous course should be repeated combined with orbital radiotherapy. An alternative approach is combination of oral or intravenous glucocorticoids with cyclosporine for 3-6 months. It was demonstrated that the combined treatment of cyclosporine with oral glucocorticoids resulted in substantial improvement and was useful to reduce the dose of glucocorticoids. In steroid unresponsive Graves’ orbitopathy, biological agents as anti-lymphocyte antibody (anti-CD 20, rituximab) and anti-cytokine antibody (anti-TNFα, etanercept) have shown promise. Theoretically somatostatin analogue pasireotide, with a wider than octreotide or lanreotide binding affinity for various somatostatin receptor subtypes could be also a treatment option. As far as the randomized clinical trials are not available, the use of biological agents and somatostatin analogs is experimental. We hope that the future treatment will be based on pathogenic mechanisms of Graves’ orbitopathy. Small molecule “drug like” TSH receptor neutral antagonists were shown to antagonize TSAb activation of TSH receptor on retroorbital preadipocytes and possibly could be used to treat Graves’ orbitopathy. Currently their effects were determined in the model of orbit cells.

